# Salidroside Modulates Insulin Signaling in a Rat Model of Nonalcoholic Steatohepatitis

**DOI:** 10.1155/2017/9651371

**Published:** 2017-02-01

**Authors:** Hongshan Li, Hao Ying, Airong Hu, Dezhou Li, Yaoren Hu

**Affiliations:** ^1^Department of Hepatology, Ningbo No. 2 Hospital, Ningbo, Zhejiang 315010, China; ^2^Medical School of Ningbo University, Ningbo, Zhejiang 315211, China

## Abstract

A growing body of evidence has shown the beneficial effects of salidroside in cardiovascular and metabolic diseases. This study aimed to evaluate the therapeutic effects of salidroside on nonalcoholic steatohepatitis (NASH) in rats and explore the underlying mechanisms related to insulin signaling. A rat model of NASH was developed by high-fat diet for 14 weeks. From week 9 onward, the treatment group received oral salidroside (4.33 mg/kg) daily for 6 weeks. Salidroside effectively attenuated steatosis and vacuolation of hepatic tissue, with a dramatic decrease in liver triglycerides and free fatty acid levels (*P* < 0.01). Dysregulation of FINS, FBG, HOMA-IR, ALT, and AST in serum was ameliorated with salidroside treatment (*P* < 0.01). In the liver, salidroside induced significant increases in key molecules in the insulin signaling pathway, such as phosphorylated insulin receptor substrate 1 (IRS1), phosphoinositide 3-kinase (PI3K), and protein kinase B (PKB), with a significant decrease in SREBP-1c levels (*P* < 0.01). Therefore, salidroside effectively protected rats from high-fat-diet-induced NASH, which may be partially attributed to its effects on the hepatic insulin signaling pathway.

## 1. Introduction

With the ever-changing environment and lifestyles, the incidence of metabolic diseases has been on the rise. Nonalcoholic fatty liver disease (NAFLD) is a pathological condition of the liver without a direct link to alcohol [1]. In addition to liver injuries, NAFLD is associated with obesity, insulin resistance, type 2 diabetes mellitus (T2DM), and metabolic syndrome. NAFLD has become the most common form of chronic liver diseases in western countries, affecting approximately 30% of the population [2]. Nonalcoholic steatohepatitis (NASH) represents a critical form of NAFLD, which may progress to hepatic fibrosis, cirrhosis, and hepatocellular carcinoma. Over the next decade, NASH is projected to be the most common indication for liver transplantation. The increasing burden of NASH worldwide has created an urgent need for the discovery of novel treatments [3].

The current treatment strategy of NASH in clinical settings is, unfortunately, limited to farnesoid X receptor (FXR) agonists (e.g., obeticholic acid) and some antidiabetic drugs (e.g., thiazolidinediones), which prove inadequate in hepatoprotection and arresting progression to more severe hepatic dysfunctions such as hepatic fibrosis [4, 5]. In the face of this challenge, traditional Chinese medicines—which have longstanding historical use in hepatoprotection and hepatic disorders—may become valuable sources for the discovery of novel drugs [6]. Salidroside, a major active component from* Rhodiola rosea *L., a traditional herbal medicine, has well known health benefits (e.g., antioxidant and antihypoxic effects) and pharmacologic properties (e.g., treatment of T2DM, cardiovascular diseases) [7–9]. In recent years, salidroside has been shown to potentially play an important role in treating hepatic injuries [10, 11]. Notably, there is some evidence that salidroside may attenuate the lipid disturbance in NASH. However, the therapeutic effect of salidroside in NASH has not been clearly established and, more importantly, the mechanism for such an effect remains elusive.

This study was conducted to evaluate the therapeutic effects of salidroside on high-fat-diet-induced nonalcoholic steatohepatitis (NASH) in rats and to explore the underlying mechanisms pertaining to insulin signaling.

## 2. Materials and Methods

### 2.1. Animals and Drug Administration

Male Sprague–Dawley rats (mean weight 100–120 g) were obtained from the Shanghai SLAC Laboratory Animal Co. Ltd. (Shanghai, China; license number SCXK-(hu)2012-0002). Animals were housed at a constant temperature (22 ± 1°C) under a 12-hour light/dark cycle, with food and water provided ad libitum. All animal experiments were approved by the Institutional Animal Care and Use Committee of Ningbo University (license number SYXK(ZHE)2013-0191).

### 2.2. High-Fat-Diet-Induced NASH and Drug Administration

Following a week of environmental habituation, the rats were randomly divided into control (*n* = 8), NASH model (*n* = 8), salidroside treatment (*n* = 8), and rosiglitazone treatment (*n* = 8) groups. NASH was induced by high-fat feeding (10% lard, 2% cholesterol, and 88% basic feed; SLAC Laboratory Animal Co. Ltd., China) for 14 consecutive weeks. Starting from the ninth week of the high-fat diet, which is routine practice when initiating treatment for NASH, rats in the salidroside and rosiglitazone treatment groups received salidroside (4.33 mg/kg; purity > 98%; Nanjing Zelang Biological Technology Co., Ltd., Nanjing, China) and rosiglitazone (0.4 mg/kg), respectively, daily through gavage. Salidroside dosage in rats was calculated by extrapolating the established human dosage per day (4.5 g of* Rhodiola rosea *L. containing ~40.9 mg of salidroside). The use of rosiglitazone in our study was supported by previous reports on its protective effects in NASH [12, 13]. Subjects in the control and model groups received the vehicle.

### 2.3. Sample Collection

At the end of week 14, the animals were fasted overnight, weighed, and then humanly euthanized. Serum samples were obtained following centrifugation of trunk blood. The liver was removed, weighed, and, after sampling for histological assessment, snap-frozen in liquid nitrogen. Both serum and liver samples were stored at −70°C until laboratory testing.

### 2.4. Analysis of Hepatic Fatty Acids and Triglycerides

Intrahepatic concentrations of triglycerides (TG) and free fatty acids (FFAs) were determined as described previously. Briefly, for FFA analysis, approximately 100 mg of hepatic tissue was homogenized, using a Bio-Gen PRO200 homogenizer, in 1 mL phosphate-buffered saline (PBS) and the homogenate was centrifuged (3600 rpm, 4°C, 20 min) to obtain a supernatant that was then used for quantitative measurements using commercially available kits (Nanjing Jiancheng Bioengineering Institute, Nanjing, China). For TG measurement, lipids in the homogenate from 200 mg of liver sample were extracted with 3 mL of ethanol-acetone (1 : 1) thrice. After overnight sedimentation, samples were centrifuged at 4°C and the supernatant was obtained for analysis using commercially available kits (Nanjing Jiancheng Bioengineering Institute, China). Briefly, 10 *μ*L of distilled water was added to the blank tube, 10 *μ*L of 2.26 mM calibrator was added to the standard quality control tube, and 10 *μ*L of sample was added to the sample tube. A total of 1000 *μ*L of working fluid was added to each of these tubes, and the solution was gently manually mixed. Then, all the tubes were kept at 37°C in a warm bath for 10 min, and optical density (OD) values were determined at a wavelength of 510 nm.

### 2.5. Serum ALT and AST Assay

Plasma alanine aminotransferase (ALT), aspartate aminotransferase (AST) activity, and fasting blood glucose levels were measured according to the manufacturer's instructions (Nanjing Jiancheng Bioengineering Institute, China). Briefly, 20 *μ*L of substrate solution was added to each of the control and assay wells in a 96-well plate, and then 5 *μ*L of each sample was added to the sample well and mixed. After 30-minute incubation at 37°C, 200 *μ*L of sodium hydroxide solution (0.4 M) was added, and the plate was then gently shaken and further incubated for 15 min at room temperature. The OD values were determined at 510 nm.

### 2.6. Biochemical Assay of Rat Plasma

Molecules involved in hepatic insulin signaling, including insulin receptor substrate 1 (IRS1), phosphorylated IRS1 (pIRS1), phosphatidylinositol 3-kinase (PI3K), phosphorylated PI3K (pPI3K), protein kinase B (PKB), phosphorylated PKB (pPKB), and hepatic sterol-regulatory element binding proteins-1c (SREBP-1c), were measured with ELISA kits (Shanghai Yuanye Bio-Technology Co., Ltd., Shanghai, China). Liver tissue homogenate was prepared as described earlier for the FFA concentration assay. Briefly, 50 *μ*L of each sample was added to the wells with precoated antibodies, and 50 *μ*L of enzyme-labeled reagent was added to each well, except for the blank well. The plate was then incubated at 37°C for 30 min. Next, 50 *μ*L of imaging solution was added, and the plate was incubated at 37°C for 15 min in the dark. Finally, the OD value was determined at 450 nm using a CYTATION 3 microplate reader (BioTek, USA) within 15 min after adding 50 *μ*L of stopping buffer. It should be noted that, to assess insulin signaling, western blot is a more reliable approach than ELISA for evaluating the levels of p-IRS and p-Akt.

### 2.7. RNA Extraction and Quantitative Real-Time PCR Analysis

Total RNA extraction from individual liver samples (*n* = 5) was undertaken with TRIzol reagent using glass beads, and the integrity was examined using optical density ratios under 260 and 280 nm. Total RNA (0.5 *µ*g) was reverse-transcribed to cDNA using a commercial kit. Real-time qPCR reactions were carried out with a SYBR®* Premix Ex Taq*™ kit in a reaction volume of 15 *µ*L. mRNA fold changes were determined using the 2^−ΔΔCT^ method using GAPDH mRNA for the calculation, and primer sequences are summarized in Table 1.

### 2.8. H&E Staining

Liver tissues (~1 cm × 1 cm × 0.5 cm) were dissected and fixed in 10% neutral buffered formalin for 24 hours and then washed with sterile water and stored in 70% ethanol. Liver tissues were embedded in paraffin, and sections (4 *μ*m thickness) were then stained with hematoxylin and eosin (H&E) for a histological study of morphology. Slices were observed under light microscopy and the degree of hepatic steatosis was determined for each sample (F0–F4). Images were obtained by the image automatic analysis system and a representative area was shown.

### 2.9. Statistical Analysis

Statistical analysis was conducted using SPSS 16.0. All data are presented as mean ± SEM. Data between groups were compared using one-way analysis of variance as well as the least significant difference test. A value of *P* < 0.05 was considered to be statistically significant.

## 3. Results

### 3.1. Salidroside Ameliorated High-Fat-Diet-Induced Hepatic Inflammation and Necrosis

**Figure 1:**
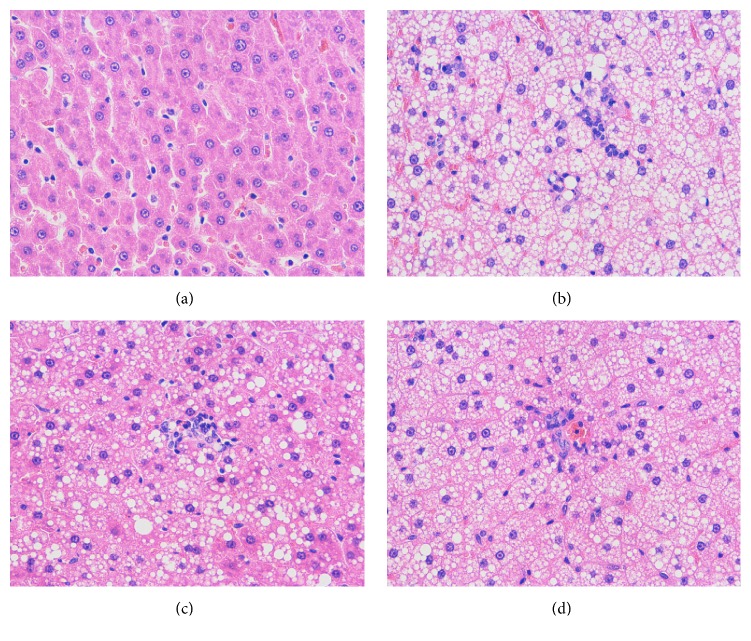
Representative liver hematoxylin and eosin staining images of rats from (a) control group, (b) model group, (c) salidroside group, and (d) rosiglitazone group, respectively. Hepatic cell degeneration was characterized by massive lipid vacuolation and diffusing necrosis. Magnification ×400.

The development of NASH was histologically evaluated using H&E staining. As hypothesized, a high-fat diet for 14 weeks induced pronounced steatosis, characterized by massive lipid vacuolation, immune cell infiltration, and diffusing necrosis. In contrast, treatment with salidroside for 6 weeks, commencing from the ninth week onward, afforded partial hepatoprotection from steatosis, as evidenced by significantly lower inflammation grades (Table 2, *P* < 0.01). Rosiglitazone, which served as a positive control, produced similar hepatoprotection to salidroside, as observed on the H&E-stained sections (Figure 1) and scores (Table 2).

### 3.2. Salidroside Decreased Lipid Dysregulation in the Liver

In addition to hepatic steatosis, a high-fat diet significantly increased hepatic TG and FFA levels (Table 3), in comparison with the control group (*P* < 0.01). Notably, salidroside and rosiglitazone effectively lowered TG and FFA levels in the test groups (*P* < 0.01), although there were no significant between-group differences among the drug treatment groups.

### 3.3. Salidroside Attenuated the Biochemical Disturbance in Rat Serum

In general, NASH is accompanied by biochemical disturbances of circulation. Accordingly, ALT and AST levels—key circulating markers of hepatic injury—were significantly higher in rats belonging to the test groups than in normal controls. Notably, salidroside could significantly lower both ALT and AST levels. However, only the ALT level showed a significant decrease in rosiglitazone-treated rats (Table 4).

As insulin resistance is a common hallmark of NASH, fasting blood glucose (FBS) and insulin levels were determined to investigate the effect of salidroside on these parameters. As shown in Table 5, there was clear evidence of insulin resistance in test rats of the NASH model as fasting insulin levels (FINS), free blood glucose (FBG), and homeostasis model assessment of insulin resistance (HOMA-IR) increased significantly. In contrast, both salidroside and rosiglitazone could alleviate these parameters of insulin resistance, suggesting effective protection from insulin resistance.

### 3.4. Salidroside Attenuated Disturbances of Hepatic Insulin Signaling

To investigate the potential mechanism underlying the effect of salidroside on NASH, insulin signaling was assessed, first, based on mRNA levels of IRS1, PI3K, and PKB in the liver. As expected, insulin signaling was impaired in the NASH model groups, and salidroside could attenuate disturbances at the mRNA level (Table 6). Meanwhile, there was only a slight, but nonsignificant, increase of these mRNA levels by rosiglitazone treatment.

Further, changes of kinase activities in the insulin signaling pathway were profiled. As shown in Table 7, insulin signaling was impaired in the model group as evidenced by impaired activation of downstream kinases. In contrast, salidroside could attenuate this impairment by significantly increasing both receptors and their phosphorylated active forms. Interestingly, there was a significant difference between salidroside and rosiglitazone groups with regard to PI3K, pPI3K, PKB, and pPKB levels.

### 3.5. Salidroside Decreased Hepatic SREBP-1c Levels in NASH Rats

Sterol-regulatory element binding proteins (SREBPs) constitute a central sensor and regulator of cholesterol levels that play an important role in hepatocyte lipid metabolism. Therefore, we further investigated hepatic mRNA and protein levels of SREBP-1c. As shown in Table 8, both mRNA and protein expression of SREBP-1c were increased in the NASH model groups compared with the control group (*P* < 0.01). In line with a powerful effect on lipid disturbance in the liver, salidroside significantly ameliorated the increase of SREBP-1c mRNA and protein (*P* < 0.05), whereas rosiglitazone could only decrease the protein expression. No significant between-group differences were detected for this effect in the 2 treatment groups.

## 4. Discussion

Despite its increasing prevalence and health impacts, there is currently no approved therapeutic agent for NASH [14]. This study aimed to investigate possible therapeutic effects and mechanisms of salidroside action in a high-fat-diet-induced NASH rat model. The results showed that salidroside treatment for 6 weeks effectively ameliorated liver injury, insulin resistance, and lipid disturbance in rats with high-fat-diet-induced NASH. These pharmacological benefits were accompanied by modulation of insulin signaling pathways in the liver, which involved IRS, PI3K, PKB, and SREBP-1c. These data not only support the notion that salidroside is a potential therapeutic agent for NASH, but also suggest that regulation of the insulin signaling pathway may represent a working strategy toward NASH treatment.

Previous studies have demonstrated that salidroside is a functionally versatile natural compound from the perennial flowering plant* Rhodiola rosea* L. [15, 16]. In addition to its benefits on the cardiovascular system, in recent years, there have been reports on metabolic improvements following administration of salidroside in animal models of obesity and diabetes. With regard to hepatic effects, previous studies have largely focused on the regulation of oxidative stress by salidroside [10, 17]. However, we show here that the protection salidroside affords against NASH may be largely derived from its modulation of signaling events downstream of insulin binding. Moreover, the regulation of SREBP-1c—a central sensor and regulator of cholesterol levels—may contribute to a large extent to its protective effects. More recently, it was appreciated that although Akt signaling is generally considered impaired in T2DM, a bidirectional relationship exists between the PI3K/Akt and SREBPs. This emerging understanding suggests that a comprehensive investigation into Akt signaling and SREBPs, as well as a balanced view on these molecules, is highly necessary in the interpretation of drug effects on NASH [18]. Therefore, our results provide a greater in-depth understanding of the hepatoprotective action of salidroside and strengthen its value in the research on anti-NASH drugs from traditional Chinese medicines.

Impairment of insulin activity is a key hallmark of NASH [19]. Our study showed that salidroside exerted effective control over the dysregulation of insulin signaling pathways related to PI3K and PKB. Specifically, salidroside significantly increased the activated status of IRS, PI3K, and PKB, which contributed to the restoration of insulin sensitivity. Of interest, a previous study in diabetic mice also reported that salidroside ameliorated insulin resistance through activation of a mitochondria-associated AMPK/PI3K/Akt/GSK3*β* pathway [20]. This finding, together with our results, suggests that the PI3K pathway downstream of insulin binding is critical to the insulin-sensitizing effects and, more broadly, metabolic benefits of salidroside. Future studies, using chemical modulators and genetic interventions of the PI3K pathway, are warranted to clarify the place of PI3K pathway in the anti-NASH effect of salidroside. Moreover, since inflammatory disturbance has a definite role in NASH and related metabolic diseases, it will be interesting to research the effects of salidroside on key inflammatory molecules in NASH rats and verify its correlation with the IRS pathway, as suggested by several recent reports on active components from herbal extracts [21–23].

In conclusion, salidroside treatment could protect rats from a high-fat-diet-induced liver injury and NASH, partially by restoring insulin responses and lipid homeostasis. Our observations demonstrated that insulin-related PI3K and PKB pathways are critical for the anti-NASH effects of salidroside. Therefore, salidroside may be a potential drug candidate that could be explored for NASH treatment. Its exact molecular mechanism and clinical application, however, require further investigation.

## Figures and Tables

**Table 1:** Summary of the primer sequences for PCR analysis.

Target genes	Sequence (5′ to 3′)
IRS1	Forward: TGGGTGGAGAGAGTATTA
	Reverse: GTGCTGTGAGGAAAGTTA
PI3K	Forward: GCCTCCATTCACCACCTCT
	Reverse: CCTCTCCTTCCAAGCCTCA
PKB	Forward: TTGTCCTTTTAGATGCTT
	Reverse: CGATTTTTATTGACTTTG
SREBP-1c	Forward: TCCTGCACCACCAACTGCTTAG
	Reverse: AGTGGCAGTGATGGCATGGACT

**Table 2:** Score of fat degeneration of liver samples on H&E staining.

Groups	Fat degeneration grading					Total
	*F*_0_	*F*_1_	*F*_2_	*F*_3_	*F*_4_	
Control	8	0	0	0	0	8^*∗∗*^
Model	0	0	0	5	3	8
Salidroside	0	1	4	3	0	8^*∗*^
Rosiglitazone	0	0	3	4	1	8^*∗*^
Total	8	1	7	12	4	32
Ridit value	0.1250	0.2656	0.3906	0.6875	0.9375	

^*∗∗*^*P* < 0.01, ^*∗*^*P* < 0.05*versus* model group.

**Table 3:** Comparison of hepatic TG and FFA levels (mean ± SEM).

Groups	*n*	TG (mg/g)	FFA (***μ***mol/g protein)
Control	8	22.03 ± 4.38^*∗∗*^	139.40 ± 13.69^*∗∗*^
Model	8	139.48 ± 25.02	668.77 ± 69.47
Salidroside	8	88.43 ± 16.04^*∗∗*^	438.27 ± 29.93^*∗∗*^
Rosiglitazone	8	103.30 ± 19.13^*∗∗*^	409.82 ± 37.29^*∗∗*^

^*∗∗*^*P* < 0.01  *versus* model group.

**Table 4:** ALT and AST parameters measured in serum (mean ± SEM).

Groups	*n*	AST (U/L)	ALT (U/L)
Control	8	21.20 ± 6.95^*∗∗*^	20.94 ± 7.49^*∗∗*^
Model	8	52.15 ± 14.10	58.55 ± 13.45
Salidroside	8	39.02 ± 9.30^*∗*^	43.95 ± 12.31^*∗∗*^
Rosiglitazone	8	45.33 ± 9.12	48.26 ± 8.27^*∗*^

^*∗∗*^*P* < 0.01, ^*∗*^*P* < 0.05*versus* model group.

**Table 5:** Serum levels of FINS and FBG and the value of HOMA-IR (mean ± SEM).

Groups	*n*	FINS (mIU/L)	FBG (mmol/L)	HOMA-IR
Control	8	16.51 ± 3.11^*∗∗*^	2.88 ± 0.27^*∗∗*^	2.10 ± 0.38^*∗∗*^
Model	8	36.20 ± 5.68	5.58 ± 0.21	8.96 ± 1.36
Salidroside	8	26.93 ± 4.88^*∗∗*^	4.31 ± 0.65^*∗∗*^	5.18 ± 1.32^*∗∗*^
Rosiglitazone	8	25.22 ± 2.98^*∗∗*^	4.22 ± 0.59^*∗∗*^	4.74 ± 0.96^*∗∗*^

^*∗∗*^*P* < 0.01  *versus* model group.

**Table 6:** Comparison of the mRNA levels of insulin signaling molecules in the liver (mean ± SEM).

Groups	*n*	IRS1	PI3K	PKB
Control	5	1.01 ± 0.13^*∗∗*^	1.02 ± 0.21^*∗∗*^	1.00 ± 0.14^*∗∗*^
Model	5	0.52 ± 0.10	0.53 ± 0.11	0.52 ± 0.11
Salidroside	5	0.72 ± 0.12^*∗*^	0.75 ± 0.15^*∗*^	0.72 ± 0.15^*∗*^
Rosiglitazone	5	0.62 ± 0.13	0.62 ± 0.12	0.63 ± 0.12

^*∗∗*^ *P* < 0.01, ^*∗*^*P* < 0.05  *versus* model group.

**Table 7:** Comparison of IRS1, pIRS1, PI3K, pPI3K, PKB, and pPKB protein levels in the liver (mean ± SEM, *n* = 8).

Groups	IRS1 (pmol/g)	pIRS1 (pmol/g)	PI3K (pmol/g)	pPI3K (pmol/g)	PKB (nmol/g)	pPKB (nmol/g)
Control	129.34 ± 24.84^*∗∗*^	55.11 ± 8.16^*∗∗*^	481.64 ± 59.49^*∗∗*^	35.51 ± 3.68^*∗∗*^	368.02 ± 38.53^*∗∗*^	161.01 ± 26.72^*∗∗*^
Model	66.22 ± 13.00	28.21 ± 4.42	239.43 ± 44.41	16.76 ± 4.21	186.41 ± 29.58	83.35 ± 16.80
Salidroside	97.63 ± 13.43^*∗∗*^	38.73 ± 5.22^*∗∗*^	328.04 ± 40.88^*∗∗*^	27.65 ± 3.62^*∗∗*^	280.31 ± 50.28^*∗∗*^	128.85 ± 10.50^*∗∗*^
Rosiglitazone	88.12 ± 15.67^*∗*^	32.70 ± 5.31	271.35 ± 30.33^#^	19.82 ± 3.69^##^	210.72 ± 40.27^##^	93.13 ± 12.62^##^

^*∗∗*^ *P* < 0.01, ^*∗*^*P* < 0.05  *versus* model group; ^##^*P* < 0.01, ^#^*P* < 0.05  *versus* salidroside group.

**Table 8:** SREBP-1c expression at the mRNA and protein level in the liver (mean ± SEM).

Groups	*N*	SREBP-1c mRNA (fold change)	*n*	SREBP-1c protein (ng/g)
Control	5	1.02 ± 0.23^*∗∗*^	8	60.22 ± 7.72^*∗∗*^
Model	5	3.28 ± 0.35	8	139.26 ± 17.53
Salidroside	5	2.56 ± 0.31^*∗∗*^	8	95.28 ± 7.51^*∗∗*^
Rosiglitazone	5	2.88 ± 0.36	8	98.57 ± 9.42^*∗∗*^

^*∗∗*^*P* < 0.01  *versus* model group.
